# Awareness and utilization of female condoms among street youths in Ibadan, an urban setting in South-West Nigeria

**DOI:** 10.11604/pamj.2019.33.168.12733

**Published:** 2019-07-04

**Authors:** Obioma Chukwudi Uchendu, Oluwapelumi Adeyera, Eme Theodora Owoaje

**Affiliations:** 1 Department of Community Medicine, Faculty of Public Health, University of Ibadan, Ibadan, Nigeria

**Keywords:** Female condoms, street youth, sexual history

## Abstract

**Introduction:**

Female condom awareness and use have been poorly documented in sub-Saharan region especially among street youths. This study assessed its awareness and use among street youths.

**Methods:**

A cross-sectional study was conducted among 964 youths between ages 15 to 24 years old using questionnaires to elicit information. Univariate and multivariate analysis were conducted at 5% level of significance.

**Results:**

More than half (69.9%) were males and between 20-25 years of age (61.2%). More than three-quarter (81.0%) had initiated sexual activity. Almost half (47.9%) of the respondents have heard about female condoms however only 16.8% have ever seen while 4.3% have actually ever used a female condom. Age, education, current sexual activity and experience of rape attempt were predictors of female condom awareness.

**Conclusion:**

Awareness of female condom was a significant predictor of utilization of female condoms. There is therefore a need for proper awareness and education on the effectiveness of female condoms.

## Introduction

Condoms are the only method available technologically that prevents against several STIs including HIV and unwanted pregnancies [1]. It has been found to be very effective compared to other forms of contraception. There are male and female condoms, however, knowledge and use of male condoms is higher than female condoms [2]. Both female and male condoms need co-operation between both partners, however, the female condom can be used in situations of male refusal to use condom [3]. The consequence of unsafe sex affects women more causing a higher prevalence HIV among women than men and unwanted pregnancy [4]. Hence, the need for a female-oriented and female-owned preventive mechanism-the female condom [4]. The female condom is currently the only effective, female-controlled (or initiated) barrier method available to prevent sexually transmitted infections (STI) including HIV and unplanned pregnancy [1, 5]. It also gives the protection and effectiveness that a male condom gives [5]. Men exert greater control over the decision to use condom, hence the advent of female condoms to give women a perceived sense of being in control [4]. It has been documented to be the best female owned prophylaxis against STIs and pregnancy [6]. Documented reasons for female condom use intention by women include: their perception of increased exposure to STI, inability to communicate their sexual and reproductive health (SRH) needs to their spouses, experiencing negative reactions after expressing their desire to uptake condom to prevent STI or pregnancy and restrained decision making concerning SRH needs [5]. According to inter-NGOs in Switzerland in 1983, street child or youth is “any girl or boy who has not reached adulthood, for whom the street (in the broadest sense of the word, including unoccupied dwellings, wasteland, etc.) has become her or his habitual abode and/or sources of livelihood, and who is inadequately protected, supervised or directed by responsible adults” [7]. Street youths are exposed to circumstances and conditions on the street which makes them susceptible to health risks and prone to developmental and emotional problems [8]. They also engage in deleterious health activities like sexual risk behaviors and substance abuse [9, 10]. Commonly reported risky sexual behaviors among street youths include non-condom use, multiple sexual partners and transactional sex [10]. They are therefore at higher risk for STIs including HIV and unwanted pregnancies [11]. Street youths have been found to initiate sex earlier and are vulnerable to sexual exploitation and abuse [8]. Furthermore, they lack information on health risk behaviors its consequences and preventive methods. They also lack access to healthcare services which predisposes them to complications of unsafe sexual behavior is also more among this set of people [9, 12] and risky behavior has also been reported among them [9, 10, 12]. Despite the peculiarities of street youths to risky sexual behaviors, most studies conducted have been focused on in-school adolescents' sexual practices and STI prevention including the knowledge and utilization of condoms in general [10], Since street youths are sexually active and indulge in unsafe sex, the female condom which is a female-initiated method of prevention enables them take decisions regarding safer sex whether consensual or not. This study therefore documented the awareness and utilization of female condoms among street youths in Ibadan, Nigeria.

## Methods

**Study Area:** this study was conducted in two local governments within Ibadan metropolis in South-West Nigeria, 90 miles north of Lagos. Ibadan is the capital of Oyo state, the largest traditional urban center in Africa and has a population of 6,393,927 as at 2011 [13]. Ibadan Municipality is made up of five Local Government Areas (LGAs) which include Ibadan North, Ibadan North East, Ibadan North West, Ibadan South East and Ibadan South West. The majority of the population are of Yoruba ethnicity and about one-quarter of the population are youths [13]. Ibadan is a major commercial city in Nigeria that with several large markets where foodstuff and other products are brought in from or taken to other parts of the country. These markets have adjoining motor parks where passengers and goods are boarded unto or from the vehicles. These areas are a beehive of activity where young people are often found till late in the night [14].

**Study design, population and sampling:** the study was a cross sectional study conducted among 964 street youths between 15 and 24 years old involved in daily street-based activities either as economical, recreational or social activities. The study participants in the study areas were identified and recruited in clusters based on the activity they were engaged in with the assistance of the community mobilisation officers in the two local government areas. These street youths were engaged in employments such as street hawking, commercial transportation, docking of goods, cart pushing, garbage scavengers and artisans [15]. A two-staged cluster sampling technique was used to select two LGA; Ibadan North (IbN LGA) and Ibadan South East (IbSE LGA). Thereafter, two market areas high in commercial and transportation activities were selected then selected from IbN LGA (Bodija and Gate) and IbSE LGA (Oranyan-Bere and Mapo). All identified areas where street youths were found were visited and consenting youths were then recruited.

**Data collection:** a semi-structured interviewer administered questionnaire was used to obtain information on socio-demographic characteristics, sexual history and awareness and utilization of female condom. Questions were adapted from the Behavioral Surveillance Surveys (BSS) [16] and existing literature [17, 18]. The instrument was translated to the local language (Yoruba) and back translated to ensure the original meanings of the questions were retained. Sixteen research assistants with secondary education were trained for two days on respecting clients' confidentiality and responses; refusal to participate in the study, not being judgmental and maintaining a neutral composure at all times. Data collection was cultural sensitive and data collectors were gender-matched.

**Data management and analysis:** each completed questionnaire was cross-checked on the spot for completeness and appropriate filling before it was further cross-checked by the two of the authors. Data was thereafter entered and analyzed using SPSS version 22 software package [19]. Descriptive statistics was presented using frequencies and percentages. Logistic regression was used to determine predictors of female condom awareness and utilization.

**Ethical considerations:** prior to the commencement of the research, ethical approval to conduct the research was obtained from the Oyo State Ethical Review Committee after an in-depth review of the proposal for compliance with ethical guidelines. Also, permission was obtained from the leaders of the identified groups where necessary. The criteria for selection of samples included voluntary declaration for participation in the study and the ability for transmission of information.

## Results

**Socio-demographics and sexual history:** there was a total of 964 street youths interviewed. More than half (69.9%) were males and between 20-25 years of age (61.2%). Few were married (13.2%) and had less than secondary school education (16.6%). Majority (85.5%) worked to earn a living but only 37.3% were currently enrolled in a school (Table 1).

**Table 1:** socio-demographic characteristics

Variable (N=964)	Frequency	Percentage (%)
Age (in years)		
15-19	374	38.8
20-25	590	61.2
Sex		
Male	674	69.9
Female	290	30.1
Marital status		
Single	834	86.5
Married in a monogamous setting	96	10.0
Married in a polygamous setting	31	3.2
Separated	3	0.3
Religion		
Islam	649	67.3
Christianity	313	32.5
Traditional	2	0.2
Tribe		
Yoruba	796	82.6
Hausa	103	10.7
Igbo	58	6.0
Others*	7	0.7
Highest educational level		
No formal education	12	1.2
Primary	148	15.4
Secondary	712	73.9
Tertiary	92	9.5
Presently enrolled in school (N=952)		
Yes	355	37.3
No	597	62.7
Work to earn a living		
Yes	824	85.5
No	140	14.5

**Awareness and utilization of female condoms:** more than three-quarter (81.0%) had initiated sexual activity, of these, 86.8% had sex in one year preceding the survey. Almost half (47.9%) of the respondents have heard about female condoms however only 16.8% have ever seen while 4.3% have actually ever used a female condom (Table 2).

**Table 2:** sexual history, awareness and utilization of female condoms

Variable (N-964)	Frequency	Percentage (%)
**Ever had sex**		
Yes	781	81.0
No	183	19.0
**Had sex in the last 12 months (N=781)**		
Yes	678	86.8
No	103	13.2
**Experienced rape attempt in last 6 months**		
Yes	99	10.3
No	865	89.7
**Ever heard about female condom**		
Yes	462	47.9
No	502	52.1
**Ever seen female condom**		
Yes	162	16.8
No	802	83.2
**Ever Used female condom**		
Yes	39	4.0
No	925	96.0

**Predictors of female condom awareness and utilization**: Table 3 shows the predictors of condom awareness and utilization. Age, education, current sexual activity and experience of rape attempt were predictors of female condom awareness. Street youths between 20-25 years and attained secondary school education or above were about 2.4 times (OR-2.365 95%CI-1.680-3.330) and 1.5 times (OR-1.509 95%CI-1.009-2.279) more likely to be aware of female condoms than their counterparts. Awareness of female condom was a significant predictor of utilization of female condom.

**Table 3:** predictors of female condom awareness and utilization

Variable (N-964)	Aware of female condom			Utilized female condom		
	Yes n (%)	No n (%)	OR (95%C.I)	Yes n (%)	No n (%)	OR (95%C.I)
Age (in years)						
15-19	131 (35.0)	243 (65.0)	2.365	7 (1.9)	367 (98.1)	1.542
20-25	331 (56.1)	259 (43.9)	(1.680-3.330)*	32 (5.4)	558 (94.6)	(0.617-3.852)
Sex						
Male	324 (48.1)	350 (51.9)	0.867	31 (4.6)	643 (95.4)	0.610
Female	138 (47.6)	152 (52.4)	(0.623-1.208)	8 (2.8)	282 (97.2)	(0.258-1.443)
Presently enrolled in school (N=952)						
Yes	150 (42.3)	205 (57.7)	1.118	13 (3.7)	342 (96.3)	0.696
No	307 (51.4)	290 (48.6)	(0.797-1.570)	25 (4.2)	572 (95.8)	(0.317-1.526)
Work to earn a living						
No	63 (45.0)	77 (55.0)	0.745	4 (2.9)	136 (97.1)	1.217
Yes	399 (48.4)	425 (51.6)	(0.462-1.201)	35 (4.2)	789 (95.8)	(0.403-3.675)
Highest educational level						
Below secondary school	61 (38.1)	99 (61.9)	1.509	5 (3.1)	155 (96.9)	1.334
Secondary school and above	401 (49.9)	403 (50.1)	(1.009-2.279)*	34 (4.2)	770 (95.8)	(0.443-4.013)
Had sex in the last 12 months (N=781)						
Yes	366 (54.0)	312 (46.0)	0.590	35 (5.2)	643 (94.8)	1.103
No	37 (35.9)	66 (64.1)	(0.376-0.925)*	4 (3.9)	99 (96.1)	(0.360-3.383)
Experienced rape attempt in last 6 months						
Yes	62 (62.6)	37 (37.4)	0.483	7 (7.1)	92 (92.9)	0.644
No	400 (46.2)	465 (53.8)	(0.300-0.777)*	32 (3.7)	833 (96.3)	(0.264-1.570)
Aware of female condom						
Yes				37 (8.0)	425 (92.0)	17.147
No				2 (0.4)	500 (99.6)	(4.061-72.400)*

* -significant variables

## Discussion

This study assessed awareness and utilization of female condoms among street youths involved in economical, recreational or social activities in market areas high in commercial and transportation activities located in Ibadan, Oyo State in Southwest Nigeria. Street youths from this study were sexually active and this is similar to findings from studies conducted in developed and developing countries as a result of lack of access to information, vulnerability to exploitation, idleness, poverty and lack of supervision or guidance [8-10, 12, 20, 21]. Awareness of condoms (47.9%) among street youths in this study was lower than what was reported among undergraduates in Edo (93.7%) and Oyo (80%) States in Nigeria but higher compared to rural dwelling women (5.2%) in Edo State [2, 18]. Undergraduate students are more likely to have access to information on prevention of STI and pregnancy including condom use either formally through their lectures or informally from their peers or internet. It is also expected that risky sexual behavior will also be reduced among them since there is some level of monitoring of their activities from their family and school authority [2]. In Cameroun, in-school young females (67.3%) reported a higher awareness of condom [22], while up to 90% of women of inner-city Denver in the United States were aware of both male and female condoms [23]. Street youths in addition to low awareness have also been reported to have low knowledge of STIs and HIV/AIDS prevention which includes condom use [10, 12]. Although utilization of female condoms in this study among street youths was low, it is not peculiar to this group alone because low utilization of female condoms have been reported in the general population. While a higher proportion of undergraduates in Oyo State reported ever using a female condom [18] compared to this study, a study in Edo State conducted among undergraduates and rural women revealed that 1.9% of undergraduate students while none of the rural dwelling women had ever used female condoms. This low female condom utilization has also been reported in Cameroon (8%) [22], Turkey (3%) [3] and U.S (5%).

Low utilization of female condoms can be attributed to; its being less well known unlike the male condom, not being readily available and accessible especially in resource poor settings [24]. Other reasons are the male-dominated cultural norms that makes it difficult for women to initiate use female (or any) condoms, the misconception of poor sexual satisfaction with the use of female or male condoms during sex preference for male condoms, partner disapproval, insertion difficulties, use of hormonal pills to prevent pregnancy, stigma and shame associated with the purchase of female condoms [2, 24, 25]. Level of education has been found to be a predictor for use of female condoms with more educated individuals utilizing it more than the less educated. This was shown in the study that compared knowledge and use of female condoms between undergraduate female students and rural dwelling women [2]. While this study reported a higher proportion of respondents with secondary education and above being aware of female condoms compared to those with primary or no education, this relationship was however significant following logistic regression. The school curriculum especially at secondary (high) school includes sexuality education and prevention of pregnancy, STIs including HIV through consistent and correct use of male and female condoms. An interesting finding from this study was that males were both more aware of and were more likely to utilize female condoms. This may not be unusual because males are more aware of condoms generally among this population group since it is easier for them to discuss prevention methods among themselves [26]. Males are more likely find it easier to buy condoms more than females because of social stigma [27]. Moreover, males would prefer sex without use of male condoms [28]. In this study however, a significant predictor for utilization of female condoms among street youths was awareness of female condoms. Street youths generally have less access to health information and sexual and reproductive health services compared to other youths [10, 21]. It is therefore necessary to make information on female condoms available to street youths and improve access to it especially among female street youths. Enhancing females' access to female condoms will reduce unequal distribution of sexual power so that women can exercise control over decision-making on safe sex. Use of appropriate social marking and promotion will enhance awareness and utilization [2, 3, 18, 25, 29].

## Conclusion

Awareness and utilization of female condoms among street youths is low. In order to achieve good sexual health outcomes for this group of people, there is a need for proper awareness and education on the effectiveness of female condoms.

### What is known about this topic

- Street youths engage in risky sexual behavior and are at a higher risk of contracting STIs including HIV compared to other adolescents;
- Awareness and utilization of female condoms among general population.

### What this study adds

- Awareness and utilization rates of female condom among street youths (vulnerable population);
- Proposes the viability of female condom intervention among female street youths to increase safe sex practices.

## Competing interests

The authors declare no competing interests.
